# In vitro assessment of the antimicrobial activity of silver and zinc oxide nanoparticles against fish pathogens

**DOI:** 10.1186/s13028-017-0317-9

**Published:** 2017-07-21

**Authors:** Mohamed Ibrahim Shaalan, Magdy Mohamed El-Mahdy, Sarah Theiner, Mansour El-Matbouli, Mona Saleh

**Affiliations:** 1 Clinical Division of Fish Medicine, University of Veterinary Medicine, Veterinärplatz 1, 1210 Vienna, Austria; 2 Department of Pathology, Faculty of Veterinary Medicine, Cairo University, Giza, 12211 Egypt; 3 Institute of Analytical Chemistry, University of Vienna, Währinger Straße 38, 1090 Vienna, Austria

**Keywords:** Antibacterial, Antifungal, Fish diseases, Silver nanoparticles, Zinc oxide nanoparticles

## Abstract

**Background:**

Antibiotic resistance is a global issue that threatens public health. The excessive use of antibiotics contributes to this problem as the genes of antibiotic resistance can be transferred between the bacteria in humans, animals and aquatic organisms. Metallic nanoparticles could serve as future substitutes for some conventional antibiotics because of their antimicrobial activity. The aim of this study was to evaluate the antimicrobial effects of silver and zinc oxide nanoparticles against major fish pathogens and assess their safety in vitro. Silver nanoparticles were synthesized by chemical reduction and characterized with UV–Vis spectroscopy, transmission electron microscopy and zeta sizer. The concentrations of silver and zinc oxide nanoparticles were measured using inductively coupled plasma-mass spectrometry. Subsequently, silver and zinc oxide nanoparticles were tested for their antimicrobial activity against *Aeromonas hydrophila*, *Aeromonas salmonicida* subsp. *salmonicida*, *Edwardsiella ictaluri, Edwardsiella tarda, Francisella noatunensis* subsp. *orientalis, Yersinia ruckeri* and *Aphanomyces invadans* and the minimum inhibitory concentrations were determined. MTT assay was performed on eel kidney cell line (EK-1) to determine the cell viability after incubation with nanoparticles. The interaction between silver nanoparticles and *A. salmonicida* was investigated by transmission electron microscopy.

**Results:**

The tested nanoparticles exhibited marked antimicrobial activity. Silver nanoparticles inhibited the growth of both *A. salmonicida* and *A. invadans* at a concentration of 17 µg/mL. Zinc oxide nanoparticles inhibited the growth of *A. salmonicida*, *Y. ruckeri* and *A. invadans* at concentrations of 15.75, 31.5 and 3.15 µg/mL respectively. Silver nanoparticles showed higher cell viability when compared to zinc oxide nanoparticles in the MTT assay. Transmission electron microscopy showed the attachment of silver nanoparticles to the bacterial membrane and disruption of its integrity.

**Conclusions:**

This is the first study on inhibitory effects of silver and zinc oxide nanoparticles towards *A. salmonicida* and *A. invadans.* Moreover, zinc oxide nanoparticles inhibited the growth of *Y. ruckeri*. In low concentrations, silver nanoparticles were less cytotoxic than zinc oxide nanoparticles and represent an alternative antimicrobial compound against *A. hydrophila*, *A. salmonicida* and *A. invadans.*

## Background

Increases in global fish consumption have resulted in greater development and intensification of aquaculture worldwide [1, 2] which have led to a massive use of antibiotics for promoting growth and prophylaxis, especially in intensive aquaculture [2]. The EU has prohibited the use of antibiotics as growth promoters in animals since 2006 [3]. However, it is difficult to assess the quantity and identify the classes of antimicrobial agents in aquaculture [4]. Tuševljak et al. [5] conducted a survey of fish farms in 25 countries in North America, South America, Europe, Africa and Asia, and found that tetracycline and quinolones are applied frequently, especially in salmon aquaculture. Nevertheless, detailed investigations about the types and the amounts of antibiotics in fish farms are still needed [4, 6]. The problem of antibiotic resistance has become a major concern in human and veterinary medicine [2, 3, 7]. The antibiotic resistance genes could be transferred between bacteria from different environments. Sharing of such genes occurs between bacteria which infect aquatic animals, terrestrial animals and humans, and thereby poses a hazard to animal and human health [7]. The closing statement of the joint science ministers meeting at the G8 summit in 2013 emphasized that antimicrobial resistance is one of the most important global health challenges in the 21st century. At the same time, bacterial, viral, mycotic and parasitic fish diseases constitute a massive threat to the aquaculture industry [8]. *Aeromonas hydrophila* is a major bacterial pathogen, which causes dermal ulceration and haemorrhagic septicemia in many fish species [9]. *Aeromonas salmonicida* was one of the first discovered causative agents of fish diseases, and is a widely-occurring pathogen of salmonids, which causes septicemia with high mortality rates [10, 11]. *Edwardsiella ictaluri* is responsible for enteric septicemia of channel catfish (*Ictalurus punctatus*), while *Edwardsiella tarda* is the causative agent of emphysematous putrefactive disease in the same species [12, 13]. *Francisella noatunensis* subsp. *orientalis* is an intracellular bacterial pathogen, which infects tilapia and produces a chronic granulomatous inflammation [14]. *Yersinia ruckeri* is the causative agent of enteric red mouth disease (ERM), which causes a wasting condition in fish and result in cumulative mortalities with high economic losses in fish farms [15, 16]. In spite of the production of a commercial vaccine against motile and non-motile strains of *Y. ruckeri*, field cases of vaccination failure have been reported [17]. Oomycete infections caused by *Aphanomyces invadans* induce skin ulcerations that extend deep into the underlying muscles leading to high morbidity and mortality in fish during outbreaks of epizootic ulcerative syndrome (EUS) [18].

Sørum [7] has reported the emergence of antibiotic resistant strains of *A. hydrophila, A. salmonicida*, and *Y. ruckeri* in fish farms. One of the recent strategies to combat microbes and multi-drug resistant bacteria is the application of metallic nanoparticles, which exhibit antimicrobial activities [19–22].

Antimicrobial effects on fish pathogens have been observed with silver and gold nanoparticles [21–28], and zinc oxide nanoparticles [29, 30].

Silver nanoparticles show antibacterial effects against *A. hydrophila, Aeromonas bestiarum, Pseudomonas flourescens, E. tarda*, *Vibrio harveyi, Proteus* spp. and *Flavobacterium* spp. [23–28, 30] and inhibit the growth of multiple drug resistant isolates of *Staphylococcus aureus*, *Micrococcus luteus* and *Klebsiella pneumonia* [31, 32]. One advantage of silver nanoparticles over conventional antibiotics is that their antimicrobial action arises through interference with multiple cellular processes of the bacteria, so the emergence of resistance is less likely [33]. Exact mechanisms that underlie the antibacterial actions of silver nanoparticles are not completely understood [34, 35]. However, modes of action have been suggested by different researchers [22, 33–36] and include the interaction of the silver nanoparticles with the bacterial cell wall, production of reactive oxygen species (ROS), interaction with DNA, and release of Ag^+^ ions.

Similarly, zinc oxide nanoparticles exhibit potent antimicrobial activities [29, 30, 37], which are suspected of arising through complex mechanisms of action that include release of Zn^2+^ ions, production of ROS and interference with bacterial replication by inhibition of cellular processes like glycolysis, acid tolerance and transmembrane proton translocation [36, 38].

In this study, we investigated the antibacterial and antifungal activity of silver and zinc oxide nanoparticles against *A. hydrophila*, *A. salmonicida* subsp. *salmonicida*, *E. ictaluri, E. tarda, F. noatunensis* subsp. *orientalis, Y. ruckeri* and *A. invadans.* We also assessed cytotoxicity and host cell viability using an MTT assay after incubating nanoparticles with eel kidney-1 cells (EK-1).

## Methods

### Materials

Silver (≈100 nm) and zinc oxide (≈66 nm) nanoparticles were purchased from Sigma-Aldrich, Austria. Chemicals and reagents used for silver nanoparticles synthesis [silver nitrate, sodium citrate tribasic hydrate, sodium borohydride, polyvinyl pyrrolidone (PVP) and de-ionized water] were also purchased from Sigma.

### Silver nanoparticles synthesis

All beakers and cylinders were thoroughly cleaned and autoclaved before use and deionized water was used for the synthesis. Silver nanoparticles synthesis was carried out by the chemical reduction method as previously described [39]. Briefly, silver nitrate was used as a source of silver, sodium borohydride solution was used as a reducing agent, and PVP acted as a stabilizing agent to prevent particles agglomeration [40]. Sodium citrate tribasic hydrate functions as both reducing and stabilizing agent at the same time, besides, the combination of PVP and sodium citrate increases the stability of the newly formed nanoparticles [41]. Silver nitrate was dissolved completely in de-ionized water then sodium citrate tribasic hydrate and PVP were added. After complete dissolving, sodium borohydride solution was added to the mixture and stirred for 30 min. The color of the solution changed from colorless to brown indicating the formation of silver nanopaticles.

As silver nanoparticles are sensitive to light, they were kept dark in a clean autoclaved bottle at 4 °C.

### Characterization of silver nanoparticles

The absorption spectra were determined using a UV–Vis spectrophotometer (NanoDrop 2000^®^, Thermo Fischer Scientific, Massachusetts, USA) against the spectrum of deionized water as a blank. All measurements were performed at room temperature on 3 separate days.

Morphology of the synthesized nanoparticles was investigated using transmission electron microscopy (EM 900, Zeiss, Oberkochen, Germany) operating at an accelerating voltage of 80 kV. One drop of silver nanoparticle solution was deposited on the carbon-coated copper grid then left to evaporate at room temperature forming a monolayer. The mean size of the particles was calculated using (Image SP Viewer^®^) software to measure 100 randomly sampled nanoparticles.

Nanoparticles size distribution and zeta potential were measured based on the dynamic light scattering (DLS) using a Malvern zeta sizer Nano ZS^®^ device. Three separate measurements on different days were performed at room temperature.

### Determination of nanoparticles concentration

Elemental concentrations of the nanoparticle solutions were measured using inductively coupled plasma-mass spectrometry (ICP-MS). Milli-Q water and nitric acid were used in all samples, with elemental standards for ICP-MS measurements from CPI international (Amsterdam, The Netherlands). Samples were digested in nitric acid using a microwave discover SP-D system (CEM Microwave Technology, Germany) with parameters: temperature 200 °C; ramp time 4 min; hold time 6 min; maximum power 300 W. Digested samples were diluted with Milli-Q water to give nitric acid concentrations lower than 3% and zinc and silver concentrations lower than 20 ng/g.

ICP-MS measurements were performed according to Theiner et al. [42] using an ICP-quadrupole MS instrument Agilent 7500ce (Agilent Technologies, Waldbronn, Germany) equipped with a CETAC ASX-520 autosampler (Nebraska, USA) and a MicroMist nebulizer, at a sample uptake rate of approx. 0.25 mL/min. The instrument was tuned daily, and rhenium served as an internal standard for zinc and silver. The ICP-MS was equipped with nickel cones and operated at an RF power of 1550 W. Argon was used as plasma gas with a flow of 15 L/min and as a carrier gas with a flow of ~1.1 L/min. The dwell time was set to 0.3 s and replicates of 10 measurements were taken. Agilent MassHunter^®^ (Workstation Software, Version B.01.01, 2012) was used for data processing.

### Bacterial strains and growth conditions

Six bacterial strains were tested: *A. hydrophila* (252/13), *A. salmonicida* subsp. *salmonicida* (A-14390), *E. ictaluri* (93/146)*, E. tarda* (30.1/14)*, F. noatunensis* subsp. *orientalis, Y. ruckeri* biotype-2 (7959-11); all were obtained from our MikroBank in the clinical division of fish medicine, University of Veterinary Medicine, Vienna, Austria. *A. hydrophila* was isolated from naturally-infected common bream (*Abramis brama* L.), *A. salmonicida* subsp. *salmonicida* and *Y. ruckeri* were isolated from naturally-infected rainbow trout (*Oncorhynchus mykiss*). *E. ictaluri* and *E. tarda* were isolated from infected channel catfish (*I. punctatus*) and discus (*Symphysodon aequifasciatus*), respectively. *F. noatunensis* subsp. *orientalis* was isolated from Malawi cichlid (*Aulonocara stuartgranti*). A loop from each pure strain was streaked on Müller-Hinton (MH) agar plates (Sigma-Aldrich) except for *F. noatunensis,* which was inoculated firstly in cystine heart broth and incubated for 24 h then streaked on cystine heart agar supplemented with 2% horse blood. All cultured agar plates were incubated at 22 °C for 24 h except for *A. salmonicida* which was incubated at 15 °C for 48 h and *F. noatunensis* which requires 5 days incubation at 22 °C for colony growth.

### Bacterial growth inhibition test

A single bacterial colony from each strain was inoculated in brain heart infusion (BHI) broth (Sigma-Aldrich) except for *F. noatunensis* which was inoculated in modified Müller-Hinton II cation-adjusted broth (Sigma-Aldrich) enriched with 2% IsoVitalex (Becton–Dickinson). After inoculation, all isolates were incubated in a shaking incubator (144 rpm) at 22 °C for 24 h, except for *A. salmonicida* which was incubated at 15 °C for 48 h. A spectrophotometer (Eppendorf BioPhotometer^®^, Eppendorf, Hamburg, Germany) was used to determine the optical density (OD_600_). Bacterial cultures were diluted with BHI broth to adjust their concentration at 10^6^ CFU/mL, which was confirmed by plate counting. Equal volumes of each nanoparticle solution and each bacterial strain were mixed to reach a final concentration of 5 × 10^5^ CFU/mL. A negative control was prepared by mixing equal volumes of bacteria and deionized water. All samples were then incubated overnight in a shaking incubator at the same conditions as above. After that, 100 µL of each sample was streaked on Müller Hinton agar plate (Sigma Aldrich) or cystine heart agar supplemented with 2% horse blood and incubated in a static incubator to observe the bacterial growth, according to Bresee et al. [43]. The test was performed in triplicate.

### Minimal inhibitory concentration (MIC)

Nanoparticles that inhibited bacterial growth were subjected to the minimal inhibitory concentration (MIC) assay in triplicate, according to Swain et al. [30] with some modifications. Double fold serial dilutions of nanoparticles were added to the cultures containing 10^6^ CFU/mL of bacteria and incubated as before. Then 100 µL of each was streaked on Müller Hinton agar plates. The double fold serial dilutions were prepared for each nanoparticle solution up to five times to determine the MIC value at which no bacterial growth on the plate was observed.

The effect of silver and zinc oxide nanoparticles on bacterial growth kinetics in BHI broth was measured using Eppendorf ^®^ spectrophotometer at 600 nm. The determined MICs of silver and zinc oxide nanoparticles were applied in this assay. Oxytetracycline (25 µg/mL) and bacterial cultures only were used as positive and negative controls, respectively. The absorbance values (OD 600) were recorded at 4, 8, 12, 16, and 24 h. The experiment was performed in triplicate.

### Fungal growth inhibition test

Silver and zinc nanoparticles were tested for their inhibitory effects against *A. invadans*. The fungus was isolated from infected dwarf gourami (*Colisa lalia*). It was grown on glucose-peptone (GP) agar and incubated at 26 °C for 5 days [18]. An anti-fungal assay was conducted as described by Mori et al. [44] with some modifications. Mycelia from the periphery of the growing fungus (about 1 mm) were inoculated in 2 mL GP broth in 10 mL falcon tubes. Equal volumes (2 mL) of each tested nanoparticle solution were added to the broth. An equal volume (2 mL) of deionized water was added to one tube as a negative control. All tubes were incubated at 26 °C for 5 days. Then, 100 µL of broth from each tube was inoculated in the center of a GP agar plate and incubated again at 26 °C for 5 days to monitor fungal growth or inhibition. Nanoparticles that inhibited the fungal growth were further diluted in twofold serial dilutions to determine the MIC. The experiment was performed in triplicate.

### Assessment of cytotoxicity via MTT assay

We assessed three concentrations of silver and zinc oxide nanoparticles for their cytotoxicity, to determine the safety of these concentrations as therapeutants in vitro. Nanoparticle concentrations were chosen based on our MIC data. Eel kidney cells (EK-1) were used in 96-well plates at approximately 1.5 × 10^4^ cells per well in L-15 medium + GlutaMAX (Gibco) supplemented with 10% fetal bovine serum (FBS) and antibiotics (Penicillin100 IU/mL and streptomycin 100 μg/mL). After 24 h incubation at 26 °C, three concentrations of silver (8.5, 17, 25.5 µg/mL) and zinc oxide nanoparticles (7.88, 15.75, 23.63 µg/mL) were added to the EK-1 cells; cells with medium alone served as negative controls. The medium was then removed and wells were washed with phosphate buffered saline (PBS) twice to remove any residual nanoparticles. Subsequently, cell viability was assessed by incubating the triplicate wells of silver and zinc oxide nanoparticles, negative control cells and blank (medium only) with MTT (3-[4,5-dimethylthiazol-2-yl]-2,5-diphenyl tetrazolium bromide, Sigma-Aldrich). MTT assay was performed as described by Mosmann [45]. 10 µL of the MTT (5 mg mL^−1^ dissolved in PBS) per 100 µL media were added to each well and incubated for 3 h at the same temperatures as described above. After that, 100 µL of MTT solubilization solution (Sigma-Aldrich) was added into each well to solubilize formazan crystals, and mixed using a rotatory shaker. The absorbance values of each well were recorded at 570 nm using EnSpire^®^ multimode plate reader. Triplicate tests were performed for each concentration and the control. After blank correction, the percentage of cell viability was calculated as the optical density values of nanoparticles-treated cells/the mean optical density of non-treated control cells ×100. Data were analyzed using one-way ANOVA test with SPSS^®^ 16.0 software.

### Ultrastructural interaction between silver nanoparticles and *A. salmonicida*

*Aeromonas. salmonicida* was incubated in BHI broth at 15 °C for 2 days, as described by Bresee et al. [43] with some modifications (Pellet was fixed with 5% glutaraldehyde in 0.1 M PBS for 4 h instead of overnight fixation with 2.5% glutaraldehyde in 0.1 M sodium cacodylate buffer). The culture was adjusted to 2.1 × 10^8^ CFU/mL, then, 1 mL added to 1 mL of silver nanoparticles (34 µg/mL), and incubated for 1 h at room temperature. The mixture was centrifuged at 11,600×*g* for 15 min, then the pellet was re-suspended in 1 mL PBS. Centrifugation was repeated with the same conditions. The pellet was fixed with 500 µL of glutaraldehyde (5% in 0.1 M PBS) at 4 °C for 4 h. Glutaraldehyde was removed and the pellet washed twice with 0.1 M PBS and incubated overnight at 4 °C. The pellet was post-fixed in 1% osmium tetroxide for 2 h at 4 °C and washed twice with PBS. Samples were dehydrated using graded alcohol series (70, 96 and 100%) before a 1:1 mixture of glycidyl ether and propylene oxide was added for 45 min then incubated overnight with a 3:1 mixture of glycidyl ether and propylene oxide. Samples were embedded in gelatin capsules then ultrathin sections were prepared using an ultramicrotome. For transmission electron microscopy (TEM) imaging, we used an EM 900 (Zeiss^®^, Oberkochen, Germany) and Image SP Viewer^®^ software.

## Results

### Characterization of silver nanoparticles

**Fig. 1:**
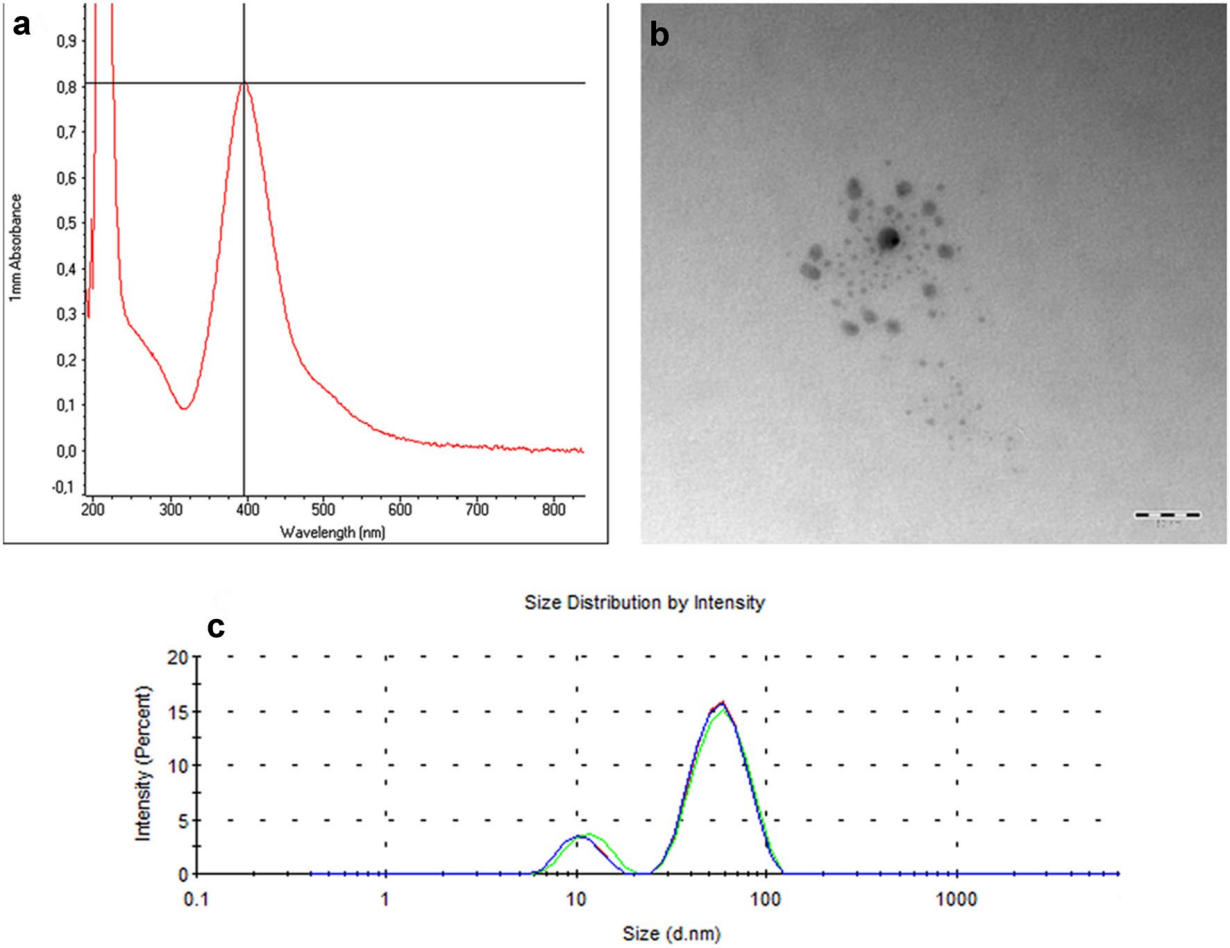
Characterization of silver nanoparticles. **a** UV–Vis analysis of silver nanoparticles showed peak absorption (0.806) at 395 nm, which matches with the surface plasmon resonance of silver nanoparticles, **b** TEM micrograph showing the morphology of silver nanoparticles: they are *spherical* with a mean size of 21 nm. (*scale bar* = 50 nm), **c** particle size distribution of the synthesized silver nanoparticles, showing two peaks: a smaller peak at 8.3 nm and a larger peak at 44.5 nm, which indicate presence of two particle size populations

UV–Vis analysis of the synthesized silver nanoparticles showed a sharp absorption peak (0.806) at 395 nm (Fig. 1a). TEM images of the synthesized silver nanoparticles showed they were spherical (Fig. 1b), with a mean diameter of 21 nm (range of sizes 11–39 nm). DLS showed two peaks: a smaller peak at 8.3 nm and a larger peak at 44.5 nm (Fig. 1c), which indicated the presence of two populations of silver nanoparticles, zeta potential value was −30.7 ± 0.45 mV.

### Determination of nanoparticles concentration

ICP-MS measurements revealed a silver concentration of 34 µg/mL in the synthesized silver nanoparticles and 16 µg/mL in the commercial silver nanoparticles. The concentration of zinc in zinc oxide nanoparticles was 63 µg/mL. Sizes and concentrations of nanoparticles are summarized in Table 1.

**Table 1:** The properties of the tested nanoparticles (sizes and concentrations)

Type of nanoparticles	Size by TEM (nm)	Size by DLS (nm)	Concentration (µg/mL)
Silver nanoparticles (synthesized)	21	Two sizes: 8.3 and 44.5	34
Silver nanoparticles (commercial)	100	96	16
Zinc oxide nanoparticles (commercial)	–	66	63

### Bacterial growth inhibition test

The synthesized silver nanoparticles and the commercial zinc oxide nanoparticles exhibited antibacterial activity against *A. hydrophila*, *A. salmonicida* and *Y. ruckeri*. However, they were not able to inhibit the growth of *E. ictaluri*, *E. tarda* and *F. noatunensis*. Commercial silver nanoparticles did not inhibit bacterial growth of the strains in this study (Table 2).

**Table 2:** Antimicrobial effects of the tested metallic nanoparticles and their minimum inhibitory concentrations (MICs) in µg/mL

Pathogen	Silver nanoparticles (synthesized) (µg/mL)	Silver nanoparticles (commercial) (µg/mL)	Zinc oxide nanoparticles (commercial) (µg/mL)
*A. hydrophila*	17	–	15.75
*A. salmonicida*	17	–	15.75
*E. ictaluri*	–	–	–
*E. tarda*	–	–	–
*F. noatunensis*	–	–	–
*Y. ruckeri*	–	–	31.5
*A. invadans*	17	–	3.15

(–) = MIC was not reached

### Minimal inhibitory concentration (MIC)

**Fig. 2:**
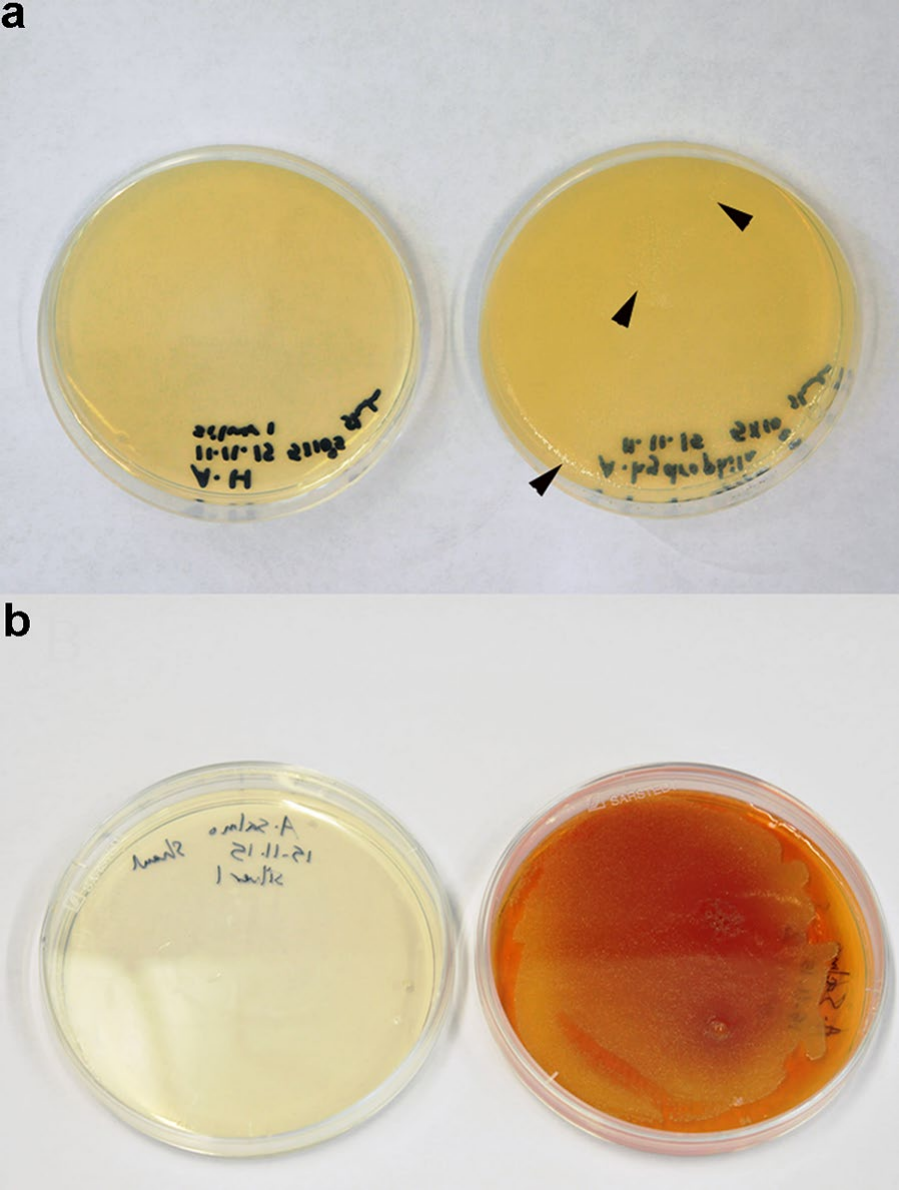
Minimum inhibitory concentration determination for silver nanoparticles. **a** Note the formation of small pinpoint colonies (*arrowheads*) of *A. hydrophila* on the control plate (*on the right*), while no bacterial growth was observed after incubation with 17 µg/mL silver nanoparticles (*on the left*), **b** growth of *A. salmonicida* with production of brown pigments on the control plate (*on the right*), while neither bacterial growth nor pigment production was observed after incubation with 17 µg/mL silver nanoparticles (*on the left*)

**Fig. 3:**
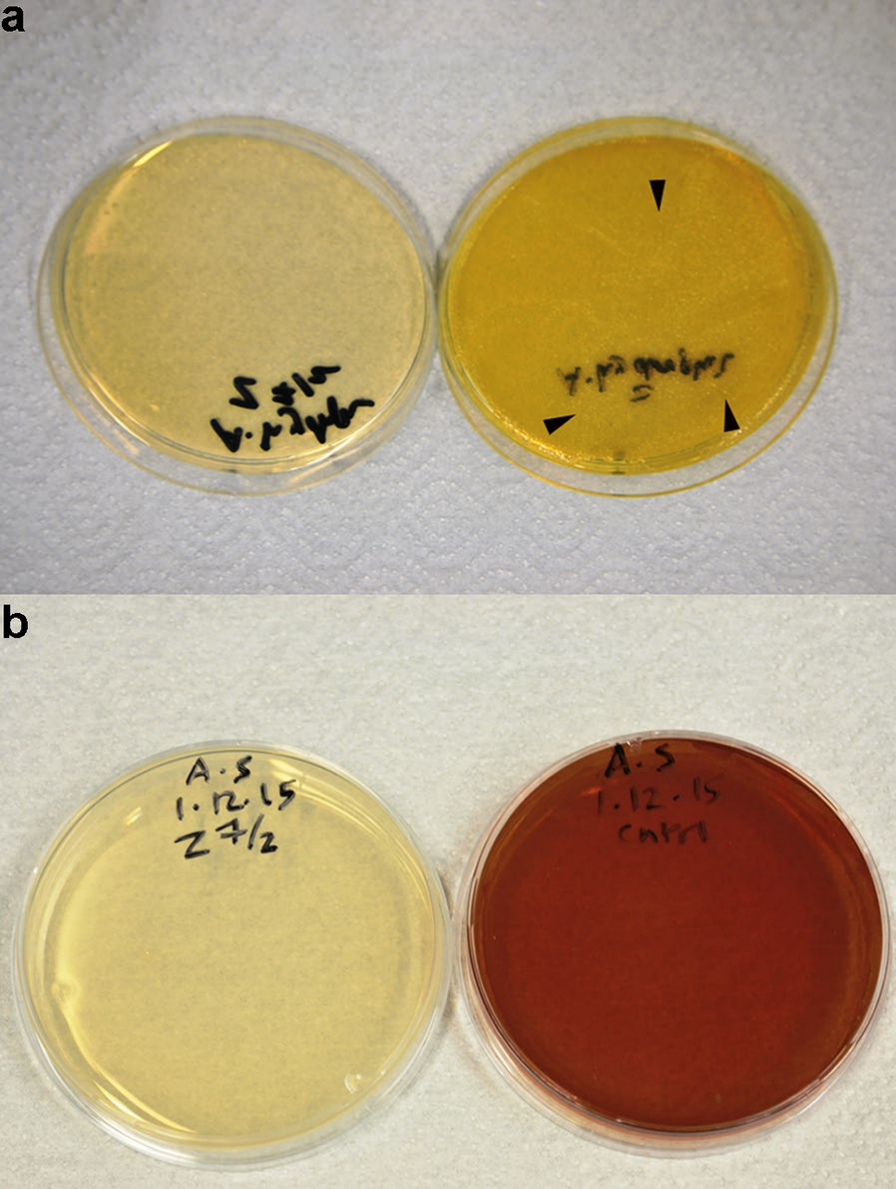
Minimum inhibitory concentration determination for zinc oxide nanoparticles. **a** Note the formation of small pinpoint colonies (*arrowheads*) of *A. hydrophila* on the control plate (*on the right*), while no bacterial growth was observed after incubation with 15.75 µg/mL zinc oxide nanoparticles (*on the left*), **b** growth of *A. salmonicida* with production of brown pigments on the control plate (*on the right*), while neither bacterial growth nor pigment production was observed after incubation with 15.75 µg/mL zinc oxide nanoparticles (*on the left*)

**Fig. 4:**
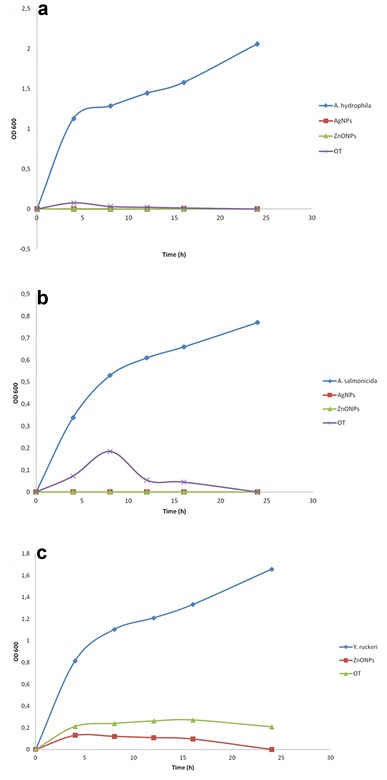
Absorbance values (OD 600) measurements for the bacterial cultures only and after incubation with silver nanoparticles, zinc oxide nanoparticles or oxytetracycline, **a** OD 600 values for *A. hydrophila*, **b** OD 600 values for *A. salmonicida*, **c** OD 600 values for *Y. ruckeri*. *AgNPs* silver nanoparticles (17 µg/mL), *ZnONPs* zinc oxide nanoparticles for *A. hydrophila* and *A. salmonicida* (15.75 µg/mL) and for *Y. ruckeri* (31.5 µg/mL), *OT* oxytetracycline (25 µg/mL)

The growth of both *A. hydrophila* and *A. salmonicida* was inhibited completely after incubation with the synthesized silver nanoparticles at 17 µg/mL or zinc oxide nanoparticles at 15.75 µg/mL. This was confirmed by the absence of bacterial growth on Müller Hinton agar plates (Figs. 2, 3), and the complete inhibition of bacterial growth in BHI as observed by a spectrophotometer (Fig. 4a, b). The growth of *Y. ruckeri* was inhibited after incubation with zinc oxide nanoparticles at 31.5 µg/mL (Table 2). That was confirmed by OD 600 measurements, which indicated complete inhibition of bacterial growth after incubation with zinc oxide nanoparticles at 24 h. In contrast, oxytetracycline (25 µg/mL) failed to reach complete inhibition of *Y. ruckeri* at the same time point (Fig. 4c).

### Fungal growth inhibition test

Silver and zinc oxide nanoparticles inhibited *A. invadans* growth in both GP broth and agar. After double fold serial dilutions, the MIC values were 17 and 3.15 µg/mL for synthesized silver and zinc oxide nanoparticles, respectively (Table 2).

### Assessment of cytotoxicity via MTT assay

Viability of EK-1 cells varied widely between silver and zinc oxide nanoparticles. The cells viability after addition 8.5 µg/mL of synthesized silver nanoparticles showed no significant difference (P ≤ 0.05) when compared with non-treated control cells. After incubation of the cells with 17 and 25.5 µg/mL of silver nanoparticles the cell viability measured 69.1 and 63% respectively (Table 3). For zinc oxide nanoparticles, all concentrations resulted in 100% cell death.

**Table 3:** Effect of different silver nanoparticles concentrations (0, 8.5, 17, 25.5 µg/mL) on EK-1 cells viability

Concentrations µg/mL	Viability %
0.0	100.3 ± 18.6^a^
8.5	92.8 ± 12.1^a^
17	69.1 ± 37.0^b^
25.5	63.0 ± 14.3^b^

The values are expressed as mean ± SD. Different letters (a, b) in the same column means that they are significantly different (P ≤ 0.05)

### Ultrastructural interaction between silver nanoparticles and *A. salmonicida*

**Fig. 5:**
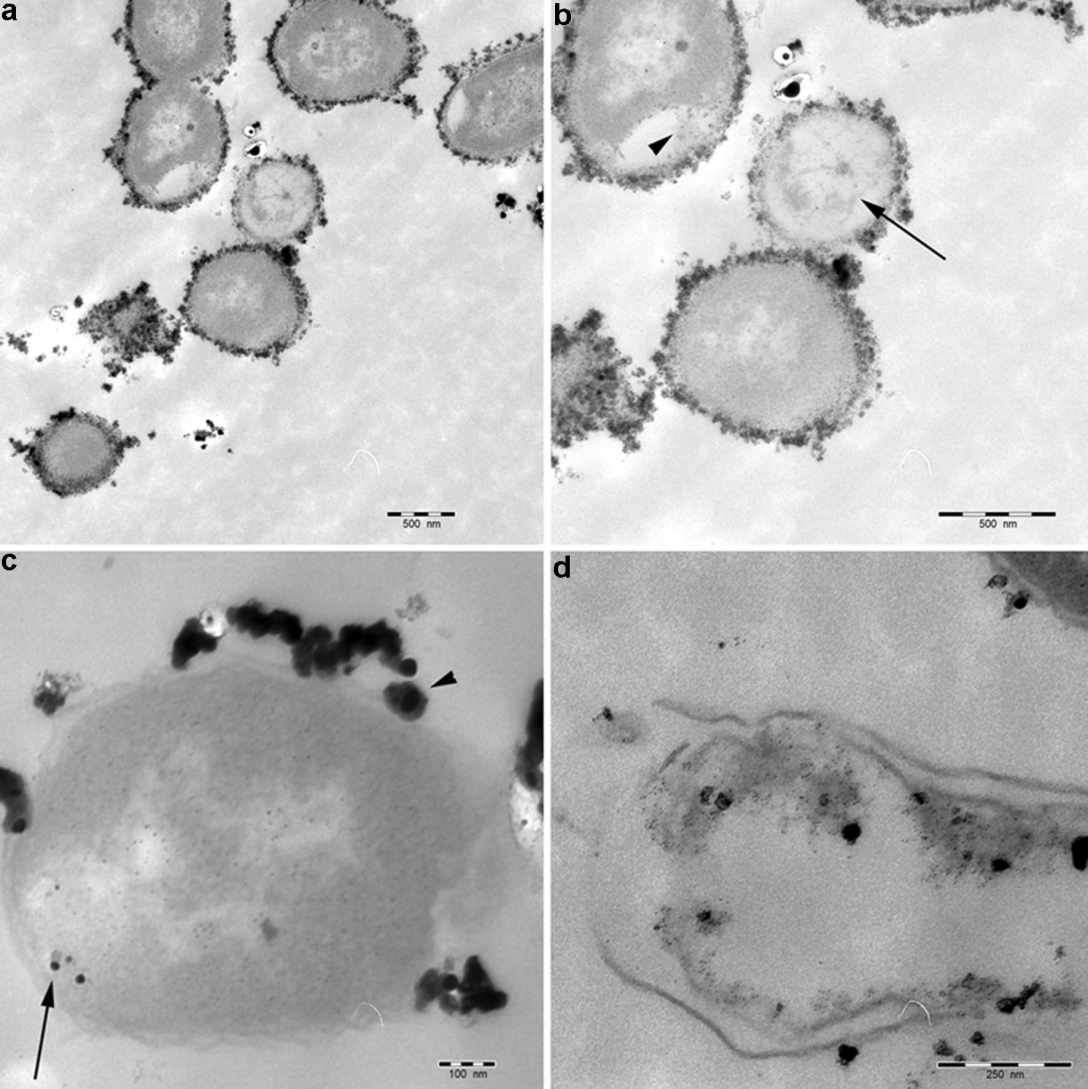
Transmission electron microscopy microphotograph showing the interaction between silver nanoparticles and *A. salmonicida*, **a** electron-dense silver nanoparticles attached to the outer membrane of *A. salmonicida* (*bar* = 500 nm). **b** Widening of periplasmic space, presence of silver nanoparticles inside the periplasm (*arrowhead*), and marked intra-cytoplasmic edema which appears electron lucent (*arrow*) (*bar* = 500 nm). **c** Silver nanoparticles attached to the outer membrane (*arrowhead*) of *A. salmonicida*, with some particles inside the bacterial cytoplasm (*arrow*) (*bar* = 100 nm). **d** Complete lysis of *A. salmonicida* by silver nanoparticles (*bar* = 250 nm)

Silver nanoparticles were observed attached to the outer membrane of *A. salmonicida* (Fig. 5a). At two-times higher magnification, we observed that silver nanoparticles induced widening of periplasmic space (arrowhead), and caused diffuse intra-cytoplasmic edema (arrow) (Fig. 5b). Smaller nanoparticles (8 nm) were observed inside the bacterial cytoplasm (arrow) (Fig. 5c). Finally, silver nanoparticles disrupted the bacterial cell membrane, leading to complete cell lysis and leakage of intracellular content (Fig. 5d).

## Discussion

Infectious diseases caused by parasites, bacteria, fungi and viruses constitute major threats to cultured fish [8]. Concurrently, there is a need to minimize use of antibiotics in farming, to combat antibiotic resistance [3, 4]. Thus, there is a growing need for alternative antimicrobial therapeutics, and metal and metal oxide nanoparticles are being widely investigated for their potential medical applications, including their use in fish medicine [22].

In this study, we investigated the antibacterial and antifungal effects of commercially obtained zinc oxide and silver nanoparticles, and synthesized silver nanoparticles in-house. For this synthesis, we used PVP as a capping agent, to stabilize the nanoparticles and prevent them from aggregating [46], with the direct benefit that PVP-capped nanoparticles are an ideal form for administration in water for fish.

UV–Vis analysis of the synthesized silver nanoparticles showed absorbance peak at 395 nm which lies in the spectrum range of silver nanoparticles and confirms the successful synthesis [47, 48]. The analysis of the size distribution of silver nanoparticles using zeta sizer revealed the presence of two peaks, one peak at 8.3 nm and a larger peak at 44.5 nm, which indicates the presence of two populations of silver nanoparticles where the majority of particles were located under the larger peak with mean size of 44.5 nm. TEM images confirmed that silver nanoparticles were spherical in shape, with an average particle size of 21 nm. While TEM provides the morphology and the mean size of the nanoparticles, zeta sizer indicates the real size distribution.

In our study, silver nanoparticles showed antibacterial activity against *A. hydrophila* which agreed with the results from previous studies [23, 25, 30]. Also, zinc oxide nanoparticles exhibited antibacterial activity against *A. hydrophila* which corresponded with Swain et al. [30]. However, in our study the MIC was measured at 15.75 µg/mL which is lower than their results which ranged from 250 to 2500 µg/mL. This difference is mostly due to the variation in the size of the nanoparticles used when comparing these two studies. The particle size along with the concentration plays a crucial role in the antibacterial properties of the nanoparticles. Smaller-sized nanoparticles have a higher surface area which increases their antibacterial activity [38].

To the best of our knowledge, this is the first study to demonstrate the inhibitory effects of silver and zinc oxide nanoparticles against *A. salmonicida* and *A. invadans,* in addition to the inhibitory effect of zinc oxide nanoparticles against *Y. ruckeri* [49].

Synthesized silver nanoparticles at a concentration of 17 µg/mL were capable of complete inhibition of the growth of both *A. salmonicida* and *A. invadans*. In comparison, oxytetracycline (25 µg/mL) required longer time to inhibit the growth of *A. salmonicida* as shown in Fig. 4b.

Using MTT assay, there was no significant difference between the lowest concentration of silver nanoparticles (8.5 µg/mL) and the control group. Silver nanoparticles were less cytotoxic to EK-1 cells when compared with zinc oxide nanoparticles. However, higher concentrations of silver nanoparticles were reported as cytotoxic and genotoxic to the fish cell lines and zebrafish [50].

Commercial silver nanoparticles failed to inhibit the bacterial growth of the tested pathogens in this study, possibly due to their lower concentration and bigger particle size in comparison with the synthesized silver nanoparticles.

Residual silver nanoparticles in the aqueous environment bind to organic and inorganic sulfur in sea or fresh water. Sulfidation of silver nanoparticles leads to significant decrease of their toxicity due to the lower solubility of the resulting silver sulfide and thus cause low detrimental impact on the environment [51].

Zinc oxide nanoparticles inhibited the growth of *A. hydrophila* and *A. salmonicida* at a concentration of 15.75 µg/mL, which is near to the MICs of silver nanoparticles.

Zinc oxide nanoparticles (31.5 µg/mL) inhibited the growth of *Y. ruckeri*, while oxytetracycline could not completely inhibit the growth of *Y. ruckeri* after 24 h (Fig. 4c).

Moreover, zinc oxide nanoparticles showed a strong antifungal activity against *A. invadans* at concentration of only 3.15 µg/mL, which was much lower than the MIC of silver nanoparticles against the same fungus. Unfortunately, the zinc oxide nanoparticles were highly toxic against the EK-1 cell line at all concentrations (7.88, 15.75, 23.63 µg/mL). These results are in accordance with Fernández et al. [52] who report high sensitivity of RTG-2, RTH-149 and RTL-W1 fish cell lines to zinc oxide nanoparticles. The observed cytotoxicity may be attributed to release of free Zn^+2^ ions [53, 54].

When zinc oxide nanoparticles are released into the aquatic environment, their behavior is controlled by water environment such as oxygen level, pH, ionic strength and amount of organic matter. Due to the high ionic strength in sea water, the zinc oxide nanoparticles tend to aggregate and become less mobile [55], while in fresh water they tend to dissolve rapidly which increases the risk of acute toxicity for aquatic organisms [56].

We investigated the interaction between silver nanoparticles and *A. salmonicida* on the ultrastructural level. TEM showed larger nanoparticles (>20 nm) bound to the bacterial cell membrane (Fig. 4), thereby presumably interfering with its integrity and function. Smaller particles (8 nm) were observed inside the bacterial cytoplasm (Fig. 4c). These observations were in concordance with previous reports [35, 57]. Presence of nanoparticles in the cytoplasm suggests that the small-sized nanoparticles could pass through the S-layer pores, which range from 2 to 8 nm in diameter as described in *A. salmonicida* [58]. In the cytoplasm, the particles would be able to react directly with the intracellular components and mechanisms.

## Conclusions

Due to their high antimicrobial activity and low environmental and cytotoxic effects, silver nanoparticles can be used as an effective antimicrobial agent against *A. hydrophila*, *A. salmonicida* and *A. invadans*. This represents a proof-of-concept for the consideration of silver nanoparticles in novel therapeutics development and disease management in aquaculture. Additional in vivo studies are needed to investigate the efficacy and safety of the silver nanoparticles in the living fish.

## References

[1] Goldburg R, Naylor R (2005). Future seascapes, fishing, and fish farming. Front Ecol Environ.

[2] Cabello FC (2008). Heavy use of prophylactic antibiotics in aquaculture: a growing problem for human and animal health and for the environment. Environ Microbiol.

[3] Menanteau-Ledouble S, Krauss I, Santos G, Fibi S, Weber B, El-Matbouli M (2015). Effect of a phytogenic feed additive on the susceptibility of *Onchorhynchus mykiss* to *Aeromonas salmonicida*. Dis Aquat Org.

[4] Cabello FC, Godfrey HP, Tomova A, Ivanova L, Dölz H, Millanao A, Buschmann AH (2013). Antimicrobial use in aquaculture re-examined: its relevance to antimicrobial resistance and to animal and human health. Environ Microbiol.

[5] Tuševljak N, Dutil L, Rajić A, Uhland FC, Mcclure C, St-Hilaire S, Reid-Smith RJ, McEwen SA (2013). Antimicrobial use and resistance in aquaculture: findings of a globally administered survey of aquaculture-allied professionals. Zoonoses Public Health.

[6] Sapkota A, Sapkota AR, Kucharski M, Burke J, Mckenzie S, Walker P, Lawrence R (2008). Aquaculture practices and potential human health risks: current knowledge and future priorities. Environ Int.

[7] Sørum H, Lie Ø (2008). Antibiotic resistance associated with veterinary drug use in fish farms. Improving farmed fish quality and safety.

[8] Meyer FP (1991). Aquaculture disease and health management. J Anim Sci.

[9] Cipriano RC, Bullock GL, Pyle SW (1984). *Aeromonas hydrophila* and motile aeromonad septicemias of fish. Fish disease leaflet 68.

[10] Austin B, Austin DA, Austin B, Austin DA (2016). *Aeromonadaceae* representative (*Aeromonas salmonicida*). Bacterial fish pathogens.

[11] Menanteau-Ledouble S, Kumar G, Saleh M, El-Matbouli M (2016). *Aeromonas salmonicida*: updates on an old acquaintance. Dis Aquat Org.

[12] Hawke JP, Mcwhorter AC, Steigerwalt AG, Brenner DJ (1981). *Edwardsiella ictaluri* sp. nov., the causative agent of enteric septicemia of catfish. Int J Sys Evol Microbiol.

[13] Meyer FP, Bullock GL (1973). *Edwardsiella tarda*, a new pathogen of channel catfish (*Ictalurus punctatus*). Appl Microbiol.

[14] Birkbeck TH, Feist SW, Verner-Jeffreys DW (2011). Francisella infections in fish and shellfish. J Fish Dis.

[15] Furones MD, Rodgers CJ, Munn CB (1993). *Yersinia ruckeri*, the causal agent of enteric redmouth disease (ERM) in fish. Annu Rev Fish Dis.

[16] Kumar G, Menanteau-Ledouble S, Saleh M, El-Matbouli M (2015). *Yersinia ruckeri*, the causative agent of enteric redmouth disease in fish. Vet Res.

[17] Huang Y, Jung A, Schäfer WJ, Mock D, Michael GB, Runge M, Schwarz S, Steinhagen D (2015). Analysis of *Yersinia ruckeri* strains isolated from trout farms in northwest Germany. Dis Aquat Org.

[18] Roberts RJ, Willoughby LG, Chinabut S (1993). Mycotic aspects of epizootic ulcerative syndrome (EUS) of Asian fishes. J Fish Dis.

[19] Huh AJ, Kwon YJ (2011). “Nanoantibiotics”: a new paradigm for treating infectious diseases using nanomaterials in the antibiotics resistant era. J Control Release.

[20] Pelgrift RY, Friedman AJ (2013). Nanotechnology as a therapeutic tool to combat microbial resistance. Adv Drug Deliv Rev.

[21] Saleh M, Kumar G, Abdel-Baki A, Al-Quraishy S, El-Matbouli M (2016). In vitro antimicrosporidial activity of gold nanoparticles against *Heterosporis saurida*. BMC Vet Res.

[22] Shaalan M, Saleh M, El-Mahdy M, El-Matbouli M (2016). Recent progress in applications of nanoparticles in fish medicine: a review. Nanomedicine.

[23] Antony JJ, Nivedheetha M, Siva D, Pradeepha G, Kokilavani P, Kalaiselvi S, Sankarganesha A, Balasundaramb A, Masilamani V, Achiraman S (2013). Antimicrobial activity of *Leucas aspera* engineered silver nanoparticles against *Aeromonas hydrophila* in infected *Catla catla*. Colloids Surf B Biointerfaces.

[24] Mahanty A, Mishra S, Bosu R, Maurya UK, Netam SP, Sarkar B (2013). Phytoextracts-synthesized silver nanoparticles inhibit bacterial fish pathogen *Aeromonas hydrophila*. Indian J Microbiol.

[25] Soltani M, Ghodratnema M, Ahari H, Ebrahimzadeh Mousavi HA, Atee M, Dastmalchi F, Rahmanya J (2009). The inhibitory effect of silver nanoparticles on the bacterial fish pathogens. *Streptococcus iniae*, *Lactococcus garvieae*, *Yersinia ruckeri* and *Aeromonas hydrophila*. Int J Vet Res.

[26] Umashankari J, Inbakandan D, Ajithkumar TT, Balasubramanian T (2012). Mangrove plant, *Rhizophora mucronata* (Lamk, 1804) mediated one pot green synthesis of silver nanoparticles and its antibacterial activity against aquatic pathogens. Aquat Biosyst.

[27] Vaseeharan B, Ramasamy P, Chen JC (2010). Antibacterial activity of silver nanoparticles (AgNps) synthesized by tea leaf extracts against pathogenic *Vibrio harveyi* and its protective efficacy on juvenile *Feneropenaeus indicus*. Lett Appl Microbiol.

[28] Velmurugan P, Iydroose M, Lee SM, Cho M, Park JH, Balachandar V, Oh BT (2014). Synthesis of silver and gold nanoparticles using cashew nut shell liquid and its antibacterial activity against fish pathogens. Indian J Microbiol.

[29] Ramamoorthy S, Kannaiyan P, Moturi M, Devadas T, Muthuramalingam J, Natarajan L, Arunachalam N, Ponniah AG (2013). Antibacterial activity of zinc oxide nanoparticles against *Vibrio harveyi*. Indian J Fish.

[30] Swain P, Nayak SK, Sasmal A, Behera T, Barik SK, Swain SK, Mishra SS, Sen AK, Das JK, Jayasankar P (2014). Antimicrobial activity of metal based nanoparticles against microbes associated with diseases in aquaculture. World J Microbiol Biotechnol.

[31] Ayala-Núñez NV, Lara HH, Turrent LDCI, Padilla CR (2009). Silver nanoparticles toxicity and bactericidal effect against methicillin resistant *Staphylococcus aureus*: nanoscale does matter. Nanobiotechnol.

[32] Prakash P, Gnanaprakasam P, Emmanuel R, Arokiyaraj S, Saravanan M (2013). Green synthesis of silver nanoparticles from leaf extract of *Mimusops elengi*, Linn. for enhanced antibacterial activity against multi drug resistant clinical isolates. Colloids Surf B Biointerfaces.

[33] Knetsch ML, Koole LH (2011). New strategies in the development of antimicrobial coatings: the example of increasing usage of silver and silver nanoparticles. Polymers.

[34] Durán N, Durán M, De Jesus MB, Seabra AB, Fávaro WJ, Nakazato G (2016). Silver nanoparticles: a new view on mechanistic aspects on antimicrobial activity. Nanomedicine.

[35] Mosselhy DA, El-Aziz MA, Hanna M, Ahmed MA, Husien MM, Feng Q (2015). Comparative synthesis and antimicrobial action of silver nanoparticles and silver nitrate. J Nanopart Res.

[36] Seil JT, Webster TJ (2012). Antimicrobial applications of nanotechnology: methods and literature. Int J Nanomed.

[37] Gunalan S, Sivaraj R, Rajendran V (2012). Green synthesized ZnO nanoparticles against bacterial and fungal pathogens. Prog Nat Sci Mater Int.

[38] Sirelkhatim A, Mahmud S, Seeni A, Kaus NHM, Ann LC, Bakhori SKM, Hasan H, Mohamad D (2015). Review on zinc oxide nanoparticles: antibacterial activity and toxicity mechanism. Nano-Micro Lett.

[39] El Mahdy MM, Eldin TAS, Aly HS, Mohammed FF, Shaalan MI (2015). Evaluation of hepatotoxic and genotoxic potential of silver nanoparticles in albino rats. Exp Toxicol Pathol.

[40] Wang H, Qiao X, Chen J, Wang X, Ding S (2005). Mechanisms of PVP in the preparation of silver nanoparticles. Mater Chem Phys.

[41] Cao VD, Tran NQ, Nguyen TPP (2015). Synergistic effect of citrate dispersant and capping polymers on controlling size growth of ultrafine copper nanoparticles. J Exp Nanosci.

[42] Theiner S, Varbanov H, Galanski M, Egger AE, Berger W, Heffeter P, Keppler BK (2014). Comparative in vitro and in vivo pharmacological investigation of platinum(IV) complexes as novel anticancer drug candidates for oral application. J Biol Inorg Chem.

[43] Bresee J, Bond CM, Worthington RJ, Smith CA, Gifford JC, Simpson CA, Carter CJ, Wang G, Hartman J, Osbaugh NA, Shoemaker RK, Melander C, Feldheim DL (2014). Nanoscale structure-activity relationships, mode of action, and biocompatibility of gold nanoparticle antibiotics. J Am Chem Soc.

[44] Mori T, Hirose H, Hanjavanit C, Hatai K (2002). Antifungal activities of plant extracts against some aquatic fungi. Biocontrol Sci.

[45] Mosmann T (1983). Rapid colorimetric assay for cellular growth and survival: application to proliferation and cytotoxicity assays. J Immunol Methods.

[46] El Badawy AME, Luxton TP, Silva RG, Scheckel KG, Suidan MT, Tolaymat TM (2010). Impact of environmental conditions (pH, ionic strength, and electrolyte type) on the surface charge and aggregation of silver nanoparticles suspensions. Environ Sci Technol.

[47] Bijanzadeh AR, Vakili MR, Khordad R (2012). A study of the surface plasmon absorption band for nanoparticles. Int J Phys Sci.

[48] Slistan-Grijalva A, Herrera-Urbina R, Rivas-Silva JF, Ávalos-Borja M, Castillón-Barraza FF, Posada-Amarillas A (2005). Classical theoretical characterization of the surface plasmon absorption band for silver spherical nanoparticles suspended in water and ethylene glycol. Physica E Low Dimens Syst Nanostruct.

[49] Shaalan M, El-Mahdy M, El-Matbouli M, Saleh M (2017). Zinc oxide nanoparticles as a novel tool to combat *Yersinia ruckeri* and *Aphanomyces invadans*. J Comp Pathol.

[50] Kim S, Ryu DY (2013). Silver nanoparticle-induced oxidative stress, genotoxicity and apoptosis in cultured cells and animal tissues. J Appl Toxicol.

[51] Levard C, Hotze EM, Lowry GV, Brown GE (2012). Environmental transformations of silver nanoparticles: impact on stability and toxicity. Environ Sci Technol.

[52] Fernández D, García-Gómez C, Babín M (2013). In vitro evaluation of cellular responses induced by ZnO nanoparticles, zinc ions and bulk ZnO in fish cells. Sci Total Environ.

[53] Brunner TJ, Wick P, Manser P, Spohn P, Grass RN, Limbach LK, Bruinink A, Stark WJ (2006). In vitro cytotoxicity of oxide nanoparticles: comparison to asbestos, silica, and the effect of particle solubility. Environ Sci Technol.

[54] Kahru A, Dubourguier HC, Blinova I, Ivask A, Kasemets K (2008). Biotests and biosensors for ecotoxicology of metal oxide nanoparticles: a minireview. Sensors.

[55] Yung MMN, Mouneyrac C, Leung KMY (2014). Ecotoxicity of zinc oxide nanoparticles in the marine environment. Encycl Nanotechnol.

[56] Franklin NM, Rogers NJ, Apte SC, Batley GE, Gadd GE, Casey PS (2007). Comparative toxicity of nanoparticulate ZnO, bulk ZnO, and ZnCl_2_ to a freshwater microalga (*Pseudokirchneriella subcapitata*): the importance of particle solubility. Environ Sci Technol.

[57] Feng QL, Wu J, Chen GQ, Cui FZ, Kim TN, Kim JO (2000). A mechanistic study of the antibacterial effect of silver ions on *Escherichia coli* and *Staphylococcus aureus*. J Biomed Mater Res.

[58] Tomás JM (2012). The main *Aeromonas* pathogenic factors. ISRN Microbiol.

